# Outcome of alcohol septal ablation in mildly symptomatic patients with hypertrophic obstructive cardiomyopathy: A comparison with medical therapy

**DOI:** 10.1002/clc.23697

**Published:** 2021-07-24

**Authors:** Peijin Li, Yuguo Xue, Jiejun Sun, Maolin Chen, Xianpeng Yu, Hua Zhao, Yuechun Gao, Xiaoling Zhang, Tengyong Jiang, Jiqiang He

**Affiliations:** ^1^ Department of Cardiology Beijing Anzhen Hospital, Beijing Institute of Heart, Lung and Blood Vessel Diseases, Capital Medical University Beijing China

**Keywords:** alcohol septal ablation, hypertrophic obstructive cardiomyopathy, mild symptom, outcome

## Abstract

**Objective:**

The aim of this study was primarily to determine efficacy after alcohol septal ablation (ASA) in mildly symptomatic patients (NYHA class II) with hypertrophic obstructive cardiomyopathy (HOCM), as compared to medical therapy.

**Methods:**

This retrospective study included 163 mildly symptomatic patients with HOCM evaluated in Beijing Anzhen Hospital between March 2001 and August 2019, consisting of the medical group (*n* = 105) and the ASA group (*n* = 58). All‐cause mortality and HCM‐related death were mainly observed.

**Results:**

Follow‐up was completed in 161 patients and the median follow‐up was 6.0 years. Compared to medically treated patients, patients post‐ASA had comparable survival free of all‐cause mortality (98.3% and 95.1% vs. 93.0% and 83.1% at 5 and 10 years, respectively; *p* = 0.374). Survival free of HCM‐related death was also similar between ASA and medical groups (98.3% and 95.1% vs. 94.3% and 86.2% at 5 and 10 years, respectively; *p* = 0.608). However, compared to medical therapy, ASA had advantages on the improvement of NYHA class (1.4 ± 0.6 vs. 2.1 ± 0.5, *p* = .000) and lower occurrence of new‐onset atrial fibrillation (AF) (7.8% vs. 20.4%, *p* = .048). Multivariate analysis demonstrated that resting LVOT gradient at the last clinical check‐up was an independent predictor of all‐cause mortality (HR = 1.021, 95%CI 1.002–1.040, *p* = .027).

**Conclusion:**

This registry suggests that mildly symptomatic patients with HOCM treated with ASA have comparable survival to that of medically treated patients, with the improvement of NYHA class and lower occurrence of new‐onset AF. All‐cause mortality is independently associated with resting LVOT gradient at the last clinical check‐up.

## 1 INTRODUCTION

Hypertrophic obstructive cardiomyopathy (HOCM) is an inherited myocardial disease caused by genetic mutations that has the important pathophysiological characteristics of marked myocardial hypertrophy (>15 mm) and dynamic left ventricular outflow tract (LVOT) obstruction.^1^, ^2^ Clinical manifestations include exertional dyspnea, chest pain, syncope and even sudden cardiac death (SCD), which seriously affect the life quality of patients.^3^ Several previous studies indicated that alcohol septal ablation (ASA) was one of the effective therapies for the relief of LVOT obstruction.^4^, ^5^, ^6^ Current guidelines recommended ASA for highly symptomatic patients (NYHA class ≥III) with HOCM.^7^ However, several data have demonstrated that markedly increased LVOT gradient has an adverse influence on the prognosis of HOCM patients irrespective of severity of clinical symptoms.^8^ In mildly symptomatic (NYHA class II) or asymptomatic patients with HOCM, this observed survival was slightly lower than expected survival of an age‐ and sex‐matched U.S. general population, and remarkably increased LVOT gradient was associated with a high risk of developing heart failure and death.^8^ Medical therapy is recommended as first‐line treatment to improve symptoms in mildly symptomatic patients with HOCM, while invasive therapy is also considered in some centers.^7^ Nevertheless, there was no definite evidence of the outcome of ASA in mildly symptomatic patients. Therefore, the purpose of this study was mainly to evaluate the efficacy of ASA compared with medical therapy in mildly symptomatic patients with HOCM, so as to provide an evidence for clinical practice.

## 2 METHODS

### 2.1 Patient selection

This retrospective study included 163 mildly symptomatic patients with HOCM evaluated in the Department of Cardiology of Beijing Anzhen Hospital affiliated to Capital Medical University between March 2001 and August 2019, consisting of the medical group (*n* = 105) and the ASA group (*n* = 58). All patients gave informed consent before enrollment. This study was approved by the Medicine Ethics Committee of Beijing Anzhen Hospital and conformed to the Declaration of Helsinki. This study was registered in the Chinese Clinical Trial Registry (No. ChiCTR2000041464).

The established diagnosis of HOCM was made by experienced cardiologists engaged in this disease, based on clinical, electrocardiographic, echocardiographic, and/or cardiac magnetic resonance imaging features, with septal hypertrophy (septal thickness ≥ 15 mm) unexplained of abnormal loading conditions.^4^, ^6^, ^8^, ^9^, ^10^ Obstruction was defined as the LVOT gradient ≥30 mmHg at rest or after provocation.^2^ Comorbidities of participants included hypertension, coronary artery disease, diabetes, chronic kidney disease and stroke. Medical group included mildly symptomatic patients who obtained sufficient symptom relief after medication (e.g., β‐blockers and verapamil). Mildly symptomatic patients met the inclusion criteria for ASA, including: (1) Intolerant to optimal medical therapy; (2) Had a strong wish for symptomatic relief; (3) LVOT gradient≥50 mmHg at rest or after provocation. Consecutive patients with the following criteria were excluded: (1) The refusal of patients; (2) Presence of severe comorbidities; (3) Presence of need for concomitant surgical procedure (e.g., coronary artery bypass grafting); (4) Septal thickness ≥ 30 mm or in the absence of appropriate coronary anatomy; (5) Presence of complete left bundle branch block; (6) The high risk of SCD, such as: family history of premature SCD, nonsustained ventricular tachycardia and documented exertional syncope. All patients were informed about the experience and potential risk of ASA at the institutional site and subsequently in agreement with the procedure, which was performed by interventional experts experienced in this disease. Details of the ASA technique have been described in several previous reports.^4^, ^11^, ^12^

### 2.2 Data collection and follow‐up

Follow‐up in the medical group was from the time registered in Beijing Anzhen Hospital to the last clinical check‐up. Follow‐up of the ASA group started at the time of procedure. Most patients had a routine clinical examination 3 months after ASA and then once every year. Check‐up of a large proportion of patients was conducted in outpatient clinic visit including the following programs: symptoms, the occurrence of clinical events, ECG, and echocardiographic parameters, while others by means of telephone contact and online communication regarding clinical data offered by local institutional sites. For patients who died outside hospitals, communication with next of kin was implemented to ascertain the cause of death.

### 2.3 Definitions and study endpoints

The primary endpoints were all‐cause mortality and HCM‐related death. In addition, the implementation of this current study in mildly symptomatic patients with HOCM was also to determine: (1) Differences in symptomatic improvement and the occurrence of new‐onset atrial fibrillation (AF) between two groups; (2) Predictors of all‐cause mortality. The causes of HCM‐related death consisted of SCD, congestive heart failure and AF‐related stroke. Periprocedural death occurring within 30 days after ASA was also considered as HCM‐related death. SCD was defined as instantaneous and unexpected death within 1 h after witnessing collapse in patients who were in a previously stable clinical condition, or nocturnal death with no antecedent history of worsening symptoms. Congestive heart failure was defined as death that occurred cardiac decompensation stage along with disease developing, and may be accompanied by pulmonary edema or cardiogenic shock.

### 2.4 Statistical analysis

Statistical analysis was conducted with SPSS 25.0 (IBM, Armonk, NY) and GraphPad (release 8.2.0; GraphPad Software Inc, La Jolla, CA). Normally distributed continuous variables were expressed as mean ± *SD*. The independent Student's *t* test was used for the comparison between two groups, while paired Student's *t* test was used within the same group. Non‐normally distributed continuous data were expressed as median (interquartile range [IQR]). The Chi square test was used to analyze categorical variables summarized as numerals (percentages). Survival analysis was performed by Kaplan–Meier method and subsequently survival difference between two groups was compared using the Log‐rank test. The prognostic predictors of clinical events were determined by Cox regression model. First in a univariable model, potential variables having an influence on the endpoint were evaluated, including: age; sex; ASA; AF at the last check‐up and some echocardiographic parameters. Second, variables with a *p* < 0.10 were entered into a backward stepwise multivariable analysis. Moreover, probability_SCD at 5 years_ was also added to Cox regression model, and the HCM Risk‐SCD formula was introduced in the 2014 ESC guidelines. All tests were two sided, and a *p* value of <0.05 was considered statistically significant.

## 3 RESULTS

### 3.1 Baseline characteristics

Baseline clinical and echocardiographic characteristics of the study population are shown in Table 1. A total of 163 patients participated in this study, including 105 patients treated with medical therapy and 58 undergoing ASA. Volume of injected alcohol during ASA was 1.9 ± 0.9 ml. Patients in the ASA group were younger (48.2 ± 11.9 years) than those in the medical group (55.4 ± 14.7 years, *p* = .002). Left atrium diameter of patients in the ASA group was larger than in the medical group (41.8 ± 5.1 vs. 39.4 ± 6.1 mm, *p* = .013). LVOT gradient at rest of patients in the ASA group was 73.5 ± 41.4 mmHg, similar to that of patients in the medical group (66.8 ± 34.7 mmHg, *p* = 0.273).

**TABLE 1:** Characteristics of 163 mildly symptomatic patients with hypertrophic obstructive cardiomyopathy (HOCM) at baseline and last clinical check‐up

	Medical therapy (*n* = 105)	ASA (*n* = 58)	*p* value
Age (years)	55.4 ± 14.7	48.2 ± 11.9	.002
Female (*n*, %)	45(42.9)	19(32.8)	0.206
BMI (kg/m^2^)	25.8 ± 3.8	26.7 ± 3.9	0.140
SBP (mmHg)	123.8 ± 16.1	127.5 ± 15.8	0.159
DBP (mmHg)	74.3 ± 10.3	77.2 ± 8.9	.072
Comorbidity (*n*, %)	58(55.2)	25(43.1)	0.138
Atrial fibrillation (*n*, %)	7(6.7)	7(12.1)	0.375
NYHA class	2.0	2.0	
Left atrium diameter (mm)	39.4 ± 6.1	41.8 ± 5.1	.013
LV end‐diastolic diameter (mm)	42.9 ± 5.0	44.0 ± 5.1	0.206
LV ejection fraction (%)	69.5 ± 7.4	68.2 ± 6.1	0.250
Septal thickness (mm)	20.3 ± 5.1	20.3 ± 4.1	0.924
Resting LVOT gradient (mmHg)	66.8 ± 34.7	73.5 ± 41.4	0.273
Medications			
Beta‐receptor antagonist (*n*, %)	83(79.0)	44(75.9)	0.639
Calcium‐channel blocker (*n*, %)	38(36.2)	16(27.6)	0.264
Follow up			
NYHA class	2.1 ± 0.5	1.4 ± 0.6^a^	.000
NYHA class I (*n*, %)	8(7.6)	38(65.5)	.000
NYHA class III/IV (*n*, %)	14(13.3)	3(5.4)	0.113
New‐onset atrial fibrillation (%)	20/98(20.4)	4/51(7.8)	.048
Left atrium diameter (mm)	43.1 ± 6.9^a^	40.9 ± 5.0	.033
LV end‐diastolic diameter (mm)	43.8 ± 4.9	45.5 ± 5.1	.042
LV ejection fraction (%)	66.3 ± 7.2^a^	64.0 ± 7.6^b^	.061
Septal thickness (mm)	19.8 ± 4.5	16.2 ± 3.5^a^	.000
Resting LVOT gradient (mmHg)	56.3 ± 27.4^a^	26.1 ± 19.1^a^	.000
Reduction in LVOT gradient (%)	28.8 ± 18.5	66.1 ± 19.3	.000

Abbreviations: ASA, alcohol septal ablation; BMI, body mass index; DBP, diastolic blood pressure; LV, left ventricular; LVOT, left ventricular outflow tract; NYHA, New York Heart Association; SBP, systolic blood pressure.

^a^ *p* < .001 compared with the baseline characteristics.

^b^ *p* < 0.01.

### 3.2 Survival analysis

**FIGURE 1:**
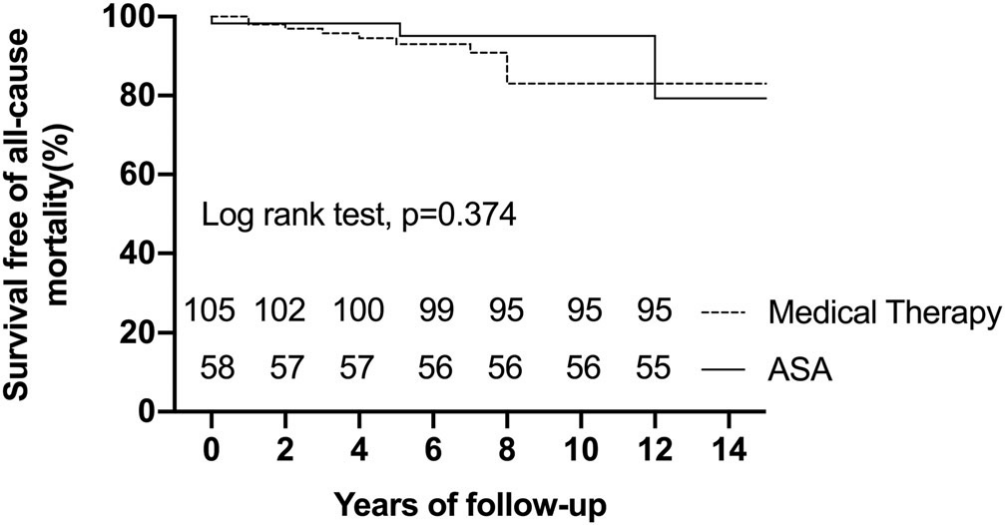
Kaplan–Meier curves depicting survival free of all‐cause mortality between the alcohol septal ablation (ASA) and medical groups

**FIGURE 2:**
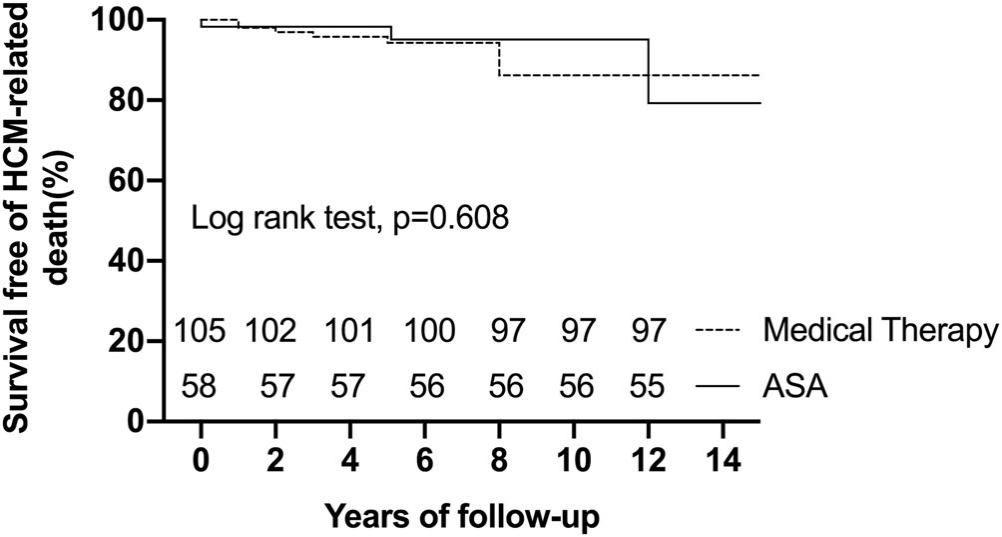
Kaplan–Meier curves depicting survival free of HCM‐related death between the alcohol septal ablation (ASA) and medical groups

During the median follow‐up of 6.0 years (IQR: 3.0–8.0 years, maximum: 18.0 years), two (1.2%) patients were lost to long‐term follow‐up. There were 14 deaths in patient cohort, including 11 deaths in the medical group and three in the ASA group, which translates into mortality rate of 0.9% per year and 0.4% per year, respectively. The classification of clinical endpoints is summarized in Table 2. Survival free of all‐cause mortality at 5 and 10 years of the ASA group was 98.3% (95%CI:88.4%–99.8%) and 95.1% (95%CI:81.0%–98.8%), respectively. This survival was comparable to that of the medical group, whose 5‐ and 10‐year survival rates were 93.0% (95%CI:84.9%–96.8%) and 83.1% (95%CI:69.5%–91.0%), respectively(*p* = 0.374) (Figure 1). The 5‐ and 10‐year survival free of HCM‐related death for two groups was 98.3% (95%CI:88.4%–99.8%) and 95.1% (95%CI:81.0%–98.8%) versus 94.3% (95%CI:86.7%–97.6%) and 86.2% (95%CI:72.7%–93.3%), respectively (*p* = 0.608) (Figure 2). SCD accounted for larger percentage (7/12, 58.3%) of HCM‐related death in this study. Three (5.1%) patients died of HCM‐related death in the ASA group, including 1(1.7%) attributable to congestive heart failure during the periprocedural period and 2(3.4%) due to SCD in the long‐term follow‐up. Cox multivariate regression analysis showed that resting LVOT gradient at the last clinical check‐up was an independent predictor of all‐cause mortality (HR =1.021, 95%CI 1.002–1.040, *p* = .027) (Table 3).

**TABLE 2:** The classification of clinical endpoints during follow‐up (*n*, %)

Events	Medical therapy (*n* = 105)	ASA (*n* = 58)	*p* value
All‐cause death	11 (10.5)	3 (5.1)	0.387
Periprocedural death		1 (1.7)	
Cardiovascular death	9 (8.6)	3 (5.1)	0.630
Noncardiovascular death	2 (1.9)	0	
HCM‐related death	9 (8.6)	3 (5.1)	0.630
Periprocedural death		1 (1.7)	
Sudden cardiac death	5 (4.8)	2 (3.4)	1.000
Congestive heart failure	1 (1.0)	1 (1.7)	1.000
AF‐related stroke	3 (2.8)	0	

**TABLE 3:** Predictors of all‐cause mortality

Variable	Univariate		Multivariate	
	HR (95%CI)	*p* value	HR (95%CI)	*p* value
Age (years)	1.037(0.994–1.082)	.096	1.034(0.990–1.081)	0.133
Female	1.625(0.542–4.870)	0.386		
ASA	0.563(0.155–2.046)	0.383		
Left atrium diameter (mm)	1.034(0.935–1.144)	0.514		
LV end‐diastolic diameter (mm)	0.951(0.856–1.057)	0.352		
Septal thickness (mm)	1.004(0.879–1.147)	0.956		
Resting LVOT gradient (mmHg)	1.009(0.996–1.023)	0.187		
Probability_SCD at 5 years_ (%)	0.957(0.696–1.316)	0.787		
Follow up				
Atrial fibrillation	1.256(0.386–4.091)	0.705		
Left atrium diameter (mm)	1.044(0.961–1.133)	0.309		
LV end‐diastolic diameter (mm)	0.903(0.803–1.014)	.084	0.935(0.830–1.053)	0.266
Septal thickness (mm)	1.080(0.972–1.200)	0.154		
Resting LVOT gradient (mmHg)	1.021(1.002–1.040)	.027	1.021(1.002–1.040)	.027

### 3.3 Clinical outcomes

Clinical and echocardiographic characteristics of the last check‐up are depicted in Table 1. ASA had reduced resting LVOT gradient from 73.5 ± 41.4 to 26.1 ± 19.1 mmHg (*p* < .001), which equates to a mean reduction of 66.1%. Septal thickness in the ASA group was reduced from 20.3 ± 4.1 to 16.2 ± 3.5 mm (*p* < .001). Moreover, the NYHA class was also remarkably improved (post‐ASA: 1.4 ± 0.6, *p*<.001), and 38(65.5%) patients were in NYHA class I. Meanwhile, obstruction was reduced from 66.8 ± 34.7 to 56.3 ± 27.4 mmHg (*p* < .001) in the medical group, which translates into a mean reduction of 28.8%. Patients of the medical group had left atrium diameter increasing from 39.4 ± 6.1 to 43.1 ± 6.9 mm (*p* < .001). When it refers to the NYHA class in the medical group, there was no statistical difference after medication (*p* = 0.202). Compared to medical therapy, ASA had advantages on the improvement of NYHA class (1.4 ± 0.6 vs. 2.1 ± 0.5, *p* = .000) and the reduction of LVOT gradient (66.1 ± 19.3% vs. 28.8 ± 18.5%, *p* = .000) at the last check‐up. By means of following up, left atrium diameter of patients in the medical group was larger than after ASA (43.1 ± 6.9 vs. 40.9 ± 5.0 mm, *p* = .033), with higher occurrence of new‐onset AF (20.4% vs. 7.8%, *p* = .048).

One (1.7%) patient was implanted an ICD for secondary prevention according to current guidelines in periprocedural phase. During the period of following up, 1(1.7%) patient underwent repeated ASA due to inadequate symptomatic relief, while 1(1.7%) patient had a permanent pacemaker implanted attributable to complete atrioventricular block 10 years after ASA. In the medical group, 2(1.9%) patients had a permanent pacemaker implanted and no patients received an ICD implantation.

## 4 DISCUSSION

This study firstly reported the efficacy of ASA used for mildly symptomatic patients with HOCM in China. Moreover, the efficacy comparison between ASA and medical groups was also revealed. The principal findings of this study are as follows: (1) Survival free of all‐cause mortality and survival free of HCM‐related death after ASA is similar to that of patients treated with medical therapy. (2) Compared to medical therapy, ASA has advantages on the improvement of NYHA class and the mean reduction of LVOT gradient, with a lower occurrence of new‐onset AF. (3) All‐cause mortality is independently associated with resting LVOT gradient at the last clinical check‐up.

Currently, ASA is predominantly recommended for highly symptomatic patients with HOCM, achieving long‐term benefits in several previous studies.^4^, ^6^, ^13^ Scarce evidence supported the clinical application of ASA procedure in asymptomatic or mildly symptomatic patients. Recently, the result was reported based on Euro‐ASA registry that long‐term survival after ASA in mildly symptomatic patients with HOCM was similar to the expected survival of an age‐ and sex‐matched general population (*p* = 0.62) and patients treated with ASA had LVOT gradient reduction and symptomatic relief with a low risk of developing heart failure.^14^ However, the perspective that positive implementation of invasive therapy in mildly symptomatic patients with HOCM has not been clear and further related research is needed. The results of the current study (mean age at ASA 48 years) demonstrate that survival free of all‐cause mortality is 98.3% at 5 years and 95.1% at 10 years after ASA, compared with the Euro‐ASA registry study^14^ (mean age at ASA 53 years) with survival rate of 94% and 87% at 5 and 10 years after ASA in mildly symptomatic patients with HOCM, respectively, and their patients are older than those included in the present study. Although overall survival of patients enrolled in our study was not compared with the age‐ and sex‐matched Chinese general population, overall survival at 5 and 10 years after ASA was higher than the survival reported by Sorajja et al.^8^ in 544 asymptomatic or mildly symptomatic patients with HOCM, who did not undergo septal reduction therapy (98.3% and 95.1% vs. 85.8% and 69.3% at 5 and 10 years, respectively). Furthermore, survival rate at 5 and 10 years after ASA in the present study was also higher than the survival reported by Veselka et al.^6^ in highly symptomatic patients treated with ASA(89.0% and 77.0% at 5 and 10 years after ASA, respectively). These findings described above may suggest the significance of timely ASA treatment for maximally improving long‐term survival in mildly symptomatic patients with HOCM intolerant to medical therapy. Different from other relevant studies, this study evaluated the efficacy difference between ASA and medical therapy in mildly symptomatic patients with HOCM. The results suggest that survival free of all‐cause mortality and survival free of HCM‐related death after ASA is comparable to that of patients in the medical group, while ASA is in a favorable position on the improvement of NYHA class and lower occurrence of new‐onset AF.

AF is the most common sustained arrhythmia in HCM, with a prevalence of 20–30%.^1^, ^15^, ^16^ The occurrence of AF as a turning point in the course of HCM leads to the impairment of life quality and the increased mortality.^17^ AF‐related stroke is one of the main causes of adverse prognosis in HCM patients.^18^ In our study, the incidence of new‐onset AF following ASA was significantly lower than that of patients in the medical group(7.8% vs. 20.4%, *p* = .048). The reason may be related to the impact of the two therapeutic methods on left atrium diameter: patients after medical therapy had a significantly increased left atrium diameter at the last check‐up, while the left atrium diameter of patients who underwent ASA was not statistically significant. It was worth noting that patients treated with ASA had larger left atrium diameter compared with patients of the medical group at baseline (41.8 ± 5.1 vs. 39.4 ± 6.1 mm, *p* = .013). Conversely, left atrium diameter of patients in the medical group was larger than after ASA at the last clinical check‐up (43.1 ± 6.9 vs. 40.9 ± 5.0 mm, *p* = .033). Several previous studies also revealed that increased left atrium diameter was independently associated with the occurrence of AF.^17^, ^19^ The possible reason why the left atrium diameter of patients undergoing ASA was not statistically significant is that ASA had a remarkable reduction of LVOT gradient compared to medical therapy (a mean reduction of 66.1% vs. 28.8%, *p* = .000). Nevertheless, whether ASA could reduce the incidence of new‐onset AF needs to be determined with a larger sample and longer follow‐up time.

Evidence from the Euro‐ASA registry indicated that LVOT gradient at the last check‐up was one of independent predictors of all‐cause mortality in highly symptomatic patients treated with ASA.^6^ Similarly, Veselka et al.^20^ suggested that residual LVOT obstruction (LVOT gradient≥30 mmHg) after ASA increased the risk of cardiovascular mortality events in highly symptomatic patients with HOCM. According to multivariable regression analysis in our study, resting LVOT gradient at the last clinical check‐up was also an independent predictor of all‐cause mortality in mildly symptomatic patients with HOCM, and each mmHg increase in LVOT gradient at the last clinical check‐up was associated with a 2.1% increase in the long‐term risk of all‐cause mortality. Therefore, the improvement of LVOT obstruction should be paid attention to during the clinical practice, regardless of the severity of symptom.

The safety of ASA procedure has been clarified in several studies.^14^, ^21^, ^22^ The rate of mortality and permanent pacemaker implantation during periprocedural period was 1.3% and 10.0%, respectively.^22^ In our current study, the rate of mortality (1.7%) during periprocedural period was similarly low compared with the result of the Euro‐ASA registry.^14^ The possible reason why the incidence of permanent pacemaker implantation was low is that volume of injected alcohol during ASA was conservative (1.9 ± 0.9 ml). Likewise, injection of large volumes of alcohol during ASA is not recommended, as it is associated with a higher incidence of permanent pacemaker dependency.^23^ In addition, scar formation after ASA is closely associated with fatal arrhythmias, and the risk of SCD is a concern. In this study, there were two patients who died of SCD in the ASA group, which was similar to the medical group.

This study was a retrospective, nonrandomized and single‐center study with a small sample, and the results were limited. First, analysis may be influenced by selection and referral bias because a part of patients from referral cannot represent the general HOCM population. Second, the efficacy and safety of ASA was cautiously extended, since patients were treated in our center experienced in HCM management in China. Third, comorbidities (e.g., coronary artery disease) were known to affect patient survival and may be more remarkable in larger sample or longer follow‐up time. This study did not evaluate the impact of relevant aspects. Fourthly, the management of AF patients, for example, indications for medical therapy including anticoagulation have changed significantly during the inclusion period. In our study, AF patients took a relatively small proportion and adhered to taking warfarin as the anticoagulant therapy. Therefore, the anticoagulant therapy considered as a confounding factor has little effect on the prognosis. Finally, overall survival of patients enrolled in our current study was not compared with expected survival of an age‐ and sex‐matched Chinese general population.

## 5 CONCLUSION

This registry suggests that mildly symptomatic patients with HOCM treated with ASA have comparable survival to that of medically treated patients. Furthermore, ASA has advantages on the improvement of NYHA class and the reduction of LVOT gradient, with a lower occurrence of new‐onset AF. Therefore, ASA could be considered as an alternative therapy for mildly symptomatic patients with HOCM intolerant to medication.

## CONFLICT OF INTEREST

The author declares there is no potential conflicts of interest.

## AUTHOR CONTRIBUTIONS

Peijin Li and Yuguo Xue are joint first authors and contributed equally to this article. Tengyong Jiang and Jiqiang He contributed equally to the design and are responsible for the article. All authors participated in the research and preparation of the manuscript.

Conceptualization: Tengyong Jiang; Jiqiang He.

Data curation: Jiejun Sun; Maolin Chen.

Formal analysis: Xianpeng Yu; Hua Zhao.

Methodology: Yuechun Gao; Xiaoling Zhang.

Roles/Writing ‐ original draft: Peijin Li; Yuguo Xue.

Writing ‐ review &editing: All.

## Data Availability

The data that support the findings of this study are available on request from the corresponding author. The data are not publicly available due to privacy or ethical restrictions.

## References

[1] Maron BJ . Clinical course and management of hypertrophic cardiomyopathy. N Engl J Med. 2018;379(20):1977. 30428294 10.1056/NEJMc1812159

[2] Batzner A , Schäfers HJ , Borisov KV , Seggewiß H . Hypertrophic obstructive cardiomyopathy. Dtsch Arztebl Int. 2019;116(4):47‐53. 30855006 10.3238/arztebl.2019.0047 PMC6415619

[3] Veselka J , Anavekar NS , Charron P . Hypertrophic obstructive cardiomyopathy. Lancet. 2017;389(10075):1253‐1267. 27912983 10.1016/S0140-6736(16)31321-6

[4] Sorajja P , Ommen SR , Holmes DR , et al. Survival after alcohol septal ablation for obstructive hypertrophic cardiomyopathy. Circulation. 2012;126(20):2374‐2380. 23076968 10.1161/CIRCULATIONAHA.111.076257

[5] Veselka J , Krejčí J , Tomašov P , et al. Long‐term survival after alcohol septal ablation for hypertrophic obstructive cardiomyopathy: a comparison with general population. Eur Heart J. 2014;35(30):2040‐2045. 24464834 10.1093/eurheartj/eht495

[6] Veselka J , Jensen MK , Liebregts M , et al. Long‐term clinical outcome after alcohol septal ablation for obstructive hypertrophic cardiomyopathy: results from the euro‐ASA registry. Eur Heart J. 2016;37(19):1517‐1523. 26746632 10.1093/eurheartj/ehv693

[7] Authors/Task Force members , Elliott PM , Anastasakis A , et al. 2014 ESC guidelines on diagnosis and management of hypertrophic cardiomyopathy: the task force for the diagnosis and Management of Hypertrophic Cardiomyopathy of the European Society of Cardiology (ESC). Eur Heart J. 2014;35(39):2733‐2779. 25173338 10.1093/eurheartj/ehu284

[8] Sorajja P , Nishimura RA , Gersh BJ , et al. Outcome of mildly symptomatic or asymptomatic obstructive hypertrophic cardiomyopathy: a long‐term follow‐up study. J Am Coll Cardiol. 2009;54(3):234‐241. 19589436 10.1016/j.jacc.2009.01.079

[9] Maron BJ , Epstein SE . Hypertrophic cardiomyopathy: a discussion of nomenclature. Am J Cardiol. 1979;43(6):1242‐1244. 571671 10.1016/0002-9149(79)90160-7

[10] Richardson P , McKenna W , Bristow M , et al. Report of the 1995 World Health Organization/international society and Federation of Cardiology Task Force on the definition and classification of cardiomyopathies. Circulation. 1996;93(5):841‐842. 8598070 10.1161/01.cir.93.5.841

[11] Sigwart U . Non‐surgical myocardial reduction for hypertrophic obstructive cardiomyopathy. Lancet. 1995;346(8969):211‐214. 7616800 10.1016/s0140-6736(95)91267-3

[12] Lakkis NM , Nagueh SF , Kleiman NS , et al. Echocardiography‐guided ethanol septal reduction for hypertrophic obstructive cardiomyopathy. Circulation. 1998;98(17):1750‐1755. 9788829 10.1161/01.cir.98.17.1750

[13] Batzner A , Pfeiffer B , Neugebauer A , Aicha D , Blank C , Seggewiss H . Survival after alcohol Septal ablation in patients with hypertrophic obstructive cardiomyopathy. J Am Coll Cardiol. 2018;72(24):3087‐3094. 30545446 10.1016/j.jacc.2018.09.064

[14] Veselka J , Faber L , Liebregts M , et al. Outcome of alcohol Septal ablation in mildly symptomatic patients with hypertrophic obstructive cardiomyopathy: a long‐term follow‐up study based on the euro‐alcohol Septal ablation registry. J Am Heart Assoc. 2017;6(5):e005735. 28512112 10.1161/JAHA.117.005735 PMC5524107

[15] Rowin EJ , Hausvater A , Link MS , et al. Clinical profile and consequences of atrial fibrillation in hypertrophic cardiomyopathy. Circulation. 2017;136(25):2420‐2436. 28916640 10.1161/CIRCULATIONAHA.117.029267

[16] Yeung C , Enriquez A , Suarez‐Fuster L , Baranchuk A . Atrial fibrillation in patients with inherited cardiomyopathies. Europace. 2019;21(1):22‐32. 29684120 10.1093/europace/euy064

[17] Zegkos T , Efthimiadis GK , Parcharidou DG , et al. Atrial fibrillation in hypertrophic cardiomyopathy: a turning point towards increased morbidity and mortality. Hellenic J Cardiol. 2017;58(5):331‐339. 28219794 10.1016/j.hjc.2017.01.027

[18] Garg L , Gupta M , Sabzwari SRA , et al. Atrial fibrillation in hypertrophic cardiomyopathy: prevalence, clinical impact, and management. Heart Fail Rev. 2019;24(2):189‐197. 30456592 10.1007/s10741-018-9752-6

[19] Liu L , Wu L , Zheng L , et al. Associations between multiple circulating biomarkers and the presence of atrial fibrillation in hypertrophic cardiomyopathy with or without left ventricular outflow tract obstruction. Int Heart J. 2019;60(2):327‐335. 30626765 10.1536/ihj.18-438

[20] Veselka J , Tomašov P , Januška J , Krejčí J , Adlová R . Obstruction after alcohol septal ablation is associated with cardiovascular mortality events. Heart. 2016;102(22):1793‐1796. 27587438 10.1136/heartjnl-2016-309699

[21] Vriesendorp PA , Liebregts M , Steggerda RC , et al. Long‐term outcomes after medical and invasive treatment in patients with hypertrophic cardiomyopathy. JACC Heart Fail. 2014;2(6):630‐636. 25447346 10.1016/j.jchf.2014.06.012

[22] Liebregts M , Vriesendorp PA , Mahmoodi BK , Schinkel AFL , Michels M , ten Berg JM . A systematic review and meta‐analysis of long‐term outcomes after Septal reduction therapy in patients with hypertrophic cardiomyopathy. JACC Heart Fail. 2015;3(11):896‐905. 26454847 10.1016/j.jchf.2015.06.011

[23] Ten Cate FJ , Soliman OI , Michels M , et al. Long‐term outcome of alcohol septal ablation in patients with obstructive hypertrophic cardiomyopathy: a word of caution. Circ Heart Fail. 2010;3(3):362‐369. 20332420 10.1161/CIRCHEARTFAILURE.109.862359

